# Supplementary material for: Pyruvate Dehydrogenase Kinase 1 inhibition mediated oxidative phosphorylation enhancement in cartilage promotes osteoarthritis progression
Source: BMC Musculoskelet Disord. 2023 Jul 20;24:597. doi: 10.1186/s12891-023-06585-6 (PMC10357736; doi:10.1186/s12891-023-06585-6)
Supplement: Supplementary file 1 — Additional file 1. [file 12891_2023_6585_MOESM1_ESM.zip › BP.Gsea.1653623667859/GOBP_HUMORAL_IMMUNE_RESPONSE_MEDIATED_BY_CIRCULATING_IMMUNOGLOBULIN.html]

Details for gene set GOBP\_HUMORAL\_IMMUNE\_RESPONSE\_MEDIATED\_BY\_CIRCULATING\_IMMUNOGLOBULIN[GSEA]

|  || Dataset | 55457\_collapsed\_to\_symbols.cls.cls #OA\_versus\_normal.cls.cls #OA\_versus\_normal\_repos |
| Phenotype | cls.cls#OA\_versus\_normal\_repos |
| Upregulated in class | OA |
| GeneSet | GOBP\_HUMORAL\_IMMUNE\_RESPONSE\_MEDIATED\_BY\_CIRCULATING\_IMMUNOGLOBULIN |
| Enrichment Score (ES) | 0.38646203 |
| Normalized Enrichment Score (NES) | 1.6920775 |
| Nominal p-value | 0.002570694 |
| FDR q-value | 0.17121501 |
| FWER p-Value | 1.0 |
Table: GSEA Results Summary

  

Fig 1: Enrichment plot: GOBP\_HUMORAL\_IMMUNE\_RESPONSE\_MEDIATED\_BY\_CIRCULATING\_IMMUNOGLOBULIN      
 Profile of the Running ES Score & Positions of GeneSet Members on the Rank Ordered List

  

| SYMBOL | TITLE | RANK IN GENE LIST | RANK METRIC SCORE | RUNNING ES | CORE ENRICHMENT || 1 | TREM2 | triggering receptor expressed on myeloid cells 2 [Source:HGNC Symbol;Acc:HGNC:17761] | 342 | 0.726 | 0.0113 | Yes |
| 2 | CFI | complement factor I [Source:HGNC Symbol;Acc:HGNC:5394] | 455 | 0.680 | 0.0381 | Yes |
| 3 | IGHG1 | immunoglobulin heavy constant gamma 1 (G1m marker) [Source:HGNC Symbol;Acc:HGNC:5525] | 607 | 0.621 | 0.0588 | Yes |
| 4 | C4A | complement C4A (Rodgers blood group) [Source:HGNC Symbol;Acc:HGNC:1323] | 632 | 0.611 | 0.0888 | Yes |
| 5 | C8B | complement C8 beta chain [Source:HGNC Symbol;Acc:HGNC:1353] | 1001 | 0.517 | 0.0872 | Yes |
| 6 | IGHV3-72 | immunoglobulin heavy variable 3-72 [Source:HGNC Symbol;Acc:HGNC:5622] | 1062 | 0.504 | 0.1088 | Yes |
| 7 | TRDC | T cell receptor delta constant [Source:HGNC Symbol;Acc:HGNC:12253] | 1100 | 0.496 | 0.1319 | Yes |
| 8 | IGLC2 | immunoglobulin lambda constant 2 [Source:HGNC Symbol;Acc:HGNC:5856] | 1210 | 0.474 | 0.1481 | Yes |
| 9 | IGHA1 | immunoglobulin heavy constant alpha 1 [Source:HGNC Symbol;Acc:HGNC:5478] | 1426 | 0.439 | 0.1543 | Yes |
| 10 | TRBC1 | T cell receptor beta constant 1 [Source:HGNC Symbol;Acc:HGNC:12156] | 1458 | 0.435 | 0.1746 | Yes |
| 11 | IGKC | immunoglobulin kappa constant [Source:HGNC Symbol;Acc:HGNC:5716] | 1477 | 0.432 | 0.1958 | Yes |
| 12 | IGHV3-33 | immunoglobulin heavy variable 3-33 [Source:HGNC Symbol;Acc:HGNC:5596] | 1511 | 0.427 | 0.2156 | Yes |
| 13 | CD55 | CD55 molecule (Cromer blood group) [Source:HGNC Symbol;Acc:HGNC:2665] | 1619 | 0.411 | 0.2287 | Yes |
| 14 | SUSD4 | sushi domain containing 4 [Source:HGNC Symbol;Acc:HGNC:25470] | 1764 | 0.389 | 0.2378 | Yes |
| 15 | MBL2 | mannose binding lectin 2 [Source:HGNC Symbol;Acc:HGNC:6922] | 1856 | 0.379 | 0.2505 | Yes |
| 16 | FCGR2B | Fc fragment of IgG receptor IIb [Source:HGNC Symbol;Acc:HGNC:3618] | 2016 | 0.360 | 0.2569 | Yes |
| 17 | CLU | clusterin [Source:HGNC Symbol;Acc:HGNC:2095] | 2083 | 0.352 | 0.2702 | Yes |
| 18 | TNF | tumor necrosis factor [Source:HGNC Symbol;Acc:HGNC:11892] | 2098 | 0.350 | 0.2873 | Yes |
| 19 | IGHM | immunoglobulin heavy constant mu [Source:HGNC Symbol;Acc:HGNC:5541] | 2241 | 0.335 | 0.2938 | Yes |
| 20 | IGHV3-21 | immunoglobulin heavy variable 3-21 [Source:HGNC Symbol;Acc:HGNC:5586] | 2404 | 0.314 | 0.2976 | Yes |
| 21 | IGHV1-69 | immunoglobulin heavy variable 1-69 [Source:HGNC Symbol;Acc:HGNC:5558] | 2461 | 0.307 | 0.3092 | Yes |
| 22 | CR2 | complement C3d receptor 2 [Source:HGNC Symbol;Acc:HGNC:2336] | 2584 | 0.292 | 0.3150 | Yes |
| 23 | IGHV4-61 | immunoglobulin heavy variable 4-61 [Source:HGNC Symbol;Acc:HGNC:5655] | 2722 | 0.278 | 0.3189 | Yes |
| 24 | C3 | complement C3 [Source:HGNC Symbol;Acc:HGNC:1318] | 2764 | 0.273 | 0.3300 | Yes |
| 25 | IGHV3-7 | immunoglobulin heavy variable 3-7 [Source:HGNC Symbol;Acc:HGNC:5620] | 2798 | 0.270 | 0.3415 | Yes |
| 26 | C1QA | complement C1q A chain [Source:HGNC Symbol;Acc:HGNC:1241] | 2902 | 0.260 | 0.3471 | Yes |
| 27 | IGHV3-73 | immunoglobulin heavy variable 3-73 [Source:HGNC Symbol;Acc:HGNC:5623] | 3082 | 0.241 | 0.3458 | Yes |
| 28 | C1QBP | complement C1q binding protein [Source:HGNC Symbol;Acc:HGNC:1243] | 3083 | 0.241 | 0.3584 | Yes |
| 29 | NOD2 | nucleotide binding oligomerization domain containing 2 [Source:HGNC Symbol;Acc:HGNC:5331] | 3154 | 0.235 | 0.3652 | Yes |
| 30 | C1QB | complement C1q B chain [Source:HGNC Symbol;Acc:HGNC:1242] | 3365 | 0.215 | 0.3601 | Yes |
| 31 | TRBC2 | T cell receptor beta constant 2 [Source:HGNC Symbol;Acc:HGNC:12157] | 3401 | 0.211 | 0.3684 | Yes |
| 32 | PTPN6 | protein tyrosine phosphatase non-receptor type 6 [Source:HGNC Symbol;Acc:HGNC:9658] | 3434 | 0.208 | 0.3767 | Yes |
| 33 | IGHV3-23 | immunoglobulin heavy variable 3-23 [Source:HGNC Symbol;Acc:HGNC:5588] | 3449 | 0.207 | 0.3865 | Yes |
| 34 | FCER2 | Fc fragment of IgE receptor II [Source:HGNC Symbol;Acc:HGNC:3612] | 3785 | 0.178 | 0.3697 | No |
| 35 | C8A | complement C8 alpha chain [Source:HGNC Symbol;Acc:HGNC:1352] | 3918 | 0.163 | 0.3679 | No |
| 36 | C7 | complement C7 [Source:HGNC Symbol;Acc:HGNC:1346] | 4078 | 0.148 | 0.3633 | No |
| 37 | ZP3 | zona pellucida glycoprotein 3 [Source:HGNC Symbol;Acc:HGNC:13189] | 4367 | 0.123 | 0.3473 | No |
| 38 | C5 | complement C5 [Source:HGNC Symbol;Acc:HGNC:1331] | 4387 | 0.122 | 0.3522 | No |
| 39 | CD46 | CD46 molecule [Source:HGNC Symbol;Acc:HGNC:6953] | 4563 | 0.108 | 0.3442 | No |
| 40 | IGHV4-34 | immunoglobulin heavy variable 4-34 [Source:HGNC Symbol;Acc:HGNC:5650] | 4746 | 0.091 | 0.3348 | No |
| 41 | CRP | C-reactive protein [Source:HGNC Symbol;Acc:HGNC:2367] | 5154 | 0.059 | 0.3062 | No |
| 42 | C9 | complement C9 [Source:HGNC Symbol;Acc:HGNC:1358] | 5415 | 0.039 | 0.2880 | No |
| 43 | IGHV5-51 | immunoglobulin heavy variable 5-51 [Source:HGNC Symbol;Acc:HGNC:5659] | 6399 | -0.035 | 0.2132 | No |
| 44 | IGHV3-20 | immunoglobulin heavy variable 3-20 [Source:HGNC Symbol;Acc:HGNC:5585] | 6859 | -0.068 | 0.1810 | No |
| 45 | SERPING1 | serpin family G member 1 [Source:HGNC Symbol;Acc:HGNC:1228] | 7286 | -0.101 | 0.1531 | No |
| 46 | C6 | complement C6 [Source:HGNC Symbol;Acc:HGNC:1339] | 7316 | -0.103 | 0.1563 | No |
| 47 | IGLL1 | immunoglobulin lambda like polypeptide 1 [Source:HGNC Symbol;Acc:HGNC:5870] | 7544 | -0.122 | 0.1449 | No |
| 48 | C1RL | complement C1r subcomponent like [Source:HGNC Symbol;Acc:HGNC:21265] | 7656 | -0.131 | 0.1431 | No |
| 49 | C1R | complement C1r [Source:HGNC Symbol;Acc:HGNC:1246] | 7716 | -0.135 | 0.1456 | No |
| 50 | PTPRC | protein tyrosine phosphatase receptor type C [Source:HGNC Symbol;Acc:HGNC:9666] | 7744 | -0.137 | 0.1507 | No |
| 51 | C4BPB | complement component 4 binding protein beta [Source:HGNC Symbol;Acc:HGNC:1328] | 7802 | -0.141 | 0.1536 | No |
| 52 | EXO1 | exonuclease 1 [Source:HGNC Symbol;Acc:HGNC:3511] | 8088 | -0.162 | 0.1399 | No |
| 53 | CD81 | CD81 molecule [Source:HGNC Symbol;Acc:HGNC:1701] | 8827 | -0.220 | 0.0939 | No |
| 54 | C8G | complement C8 gamma chain [Source:HGNC Symbol;Acc:HGNC:1354] | 8871 | -0.223 | 0.1022 | No |
| 55 | FOXJ1 | forkhead box J1 [Source:HGNC Symbol;Acc:HGNC:3816] | 9364 | -0.261 | 0.0775 | No |
| 56 | IGHD | immunoglobulin heavy constant delta [Source:HGNC Symbol;Acc:HGNC:5480] | 9396 | -0.263 | 0.0888 | No |
| 57 | C4BPA | complement component 4 binding protein alpha [Source:HGNC Symbol;Acc:HGNC:1325] | 9505 | -0.274 | 0.0948 | No |
| 58 | LTA | lymphotoxin alpha [Source:HGNC Symbol;Acc:HGNC:6709] | 9921 | -0.313 | 0.0788 | No |
| 59 | C1S | complement C1s [Source:HGNC Symbol;Acc:HGNC:1247] | 10012 | -0.323 | 0.0887 | No |
| 60 | APCS | "amyloid P component, serum [Source:HGNC Symbol;Acc:HGNC:584]" | 10326 | -0.353 | 0.0827 | No |
| 61 | IGHE | immunoglobulin heavy constant epsilon [Source:HGNC Symbol;Acc:HGNC:5522] | 10676 | -0.390 | 0.0760 | No |
| 62 | MASP2 | MBL associated serine protease 2 [Source:HGNC Symbol;Acc:HGNC:6902] | 11001 | -0.430 | 0.0732 | No |
| 63 | HPX | hemopexin [Source:HGNC Symbol;Acc:HGNC:5171] | 11156 | -0.451 | 0.0847 | No |
| 64 | CR1 | complement C3b/C4b receptor 1 (Knops blood group) [Source:HGNC Symbol;Acc:HGNC:2334] | 11376 | -0.483 | 0.0929 | No |
| 65 | BCL3 | BCL3 transcription coactivator [Source:HGNC Symbol;Acc:HGNC:998] | 11431 | -0.493 | 0.1144 | No |
Table: GSEA details [plain text format]

  

Fig 2: GOBP\_HUMORAL\_IMMUNE\_RESPONSE\_MEDIATED\_BY\_CIRCULATING\_IMMUNOGLOBULIN      
 Blue-Pink O' Gram in the Space of the Analyzed GeneSet

  

Fig 3: GOBP\_HUMORAL\_IMMUNE\_RESPONSE\_MEDIATED\_BY\_CIRCULATING\_IMMUNOGLOBULIN: Random ES distribution      
 Gene set null distribution of ES for **GOBP\_HUMORAL\_IMMUNE\_RESPONSE\_MEDIATED\_BY\_CIRCULATING\_IMMUNOGLOBULIN**
